# Preoperative proton pump inhibitor therapy and its influence on postoperative complications following major liver resection

**DOI:** 10.1007/s00423-026-04119-x

**Published:** 2026-06-27

**Authors:** Lukas Pollmann, Aladdin Ali Deeb, Falk Rauchfuß, Felix Dondorf, Nicola S. Pollmann, Utz Settmacher

**Affiliations:** Department of General-, Visceral- and Vascular Surgery, University Hospital Jena, Am Klinikum 1, Jena, 07747 Germany

**Keywords:** Proton pump inhibitors, Biliary leakage, Major liver resection, Postoperative morbidity

## Abstract

**Purpose:**

Proton pump inhibitors (PPIs) are frequently used perioperatively in patients undergoing major liver resection, although their impact on postoperative morbidity remains unclear. This study evaluated the association between preoperative PPI therapy and postoperative complications, particularly biliary leakage.

**Methods:**

This retrospective single-center cohort study included patients undergoing major liver resection (≥ 4 Couinaud segments) between 2006 and 2024. Patients with and without preoperative PPI therapy were compared regarding postoperative morbidity, assessed using the Comprehensive Complication Index (CCI), and biliary leakage.

**Results:**

A total of 507 patients were included, of whom 220 (43.4%) received preoperative PPI therapy. Patients receiving PPI therapy had a significantly higher postoperative CCI compared with those without PPI therapy (24.4 ± 25 vs. 17.7 ± 19.7, *p* = 0.002). Biliary leakage occurred more frequently in the PPI group (38% vs. 26%, *p* = 0.009), including after propensity score matching. In multivariable logistic regression analysis, preoperative PPI therapy remained independently associated with postoperative biliary leakage (OR 1.95, 95% CI 1.21–3.14; *p* = 0.006). Microbiological analysis of biliary swabs showed no relevant differences in pathogen profiles between groups.

**Conclusions:**

Preoperative PPI therapy was associated with increased postoperative morbidity and biliary leakage after major liver resection, even after adjustment for baseline risk factors. Given the observational design, these findings should be considered hypothesis-generating and support careful evaluation of perioperative PPI use in patients without a clear indication.

**Trial registration:**

The study was registered in the German Clinical Trials Register (application number DRKS00038981).

**Graphical Abstract:**

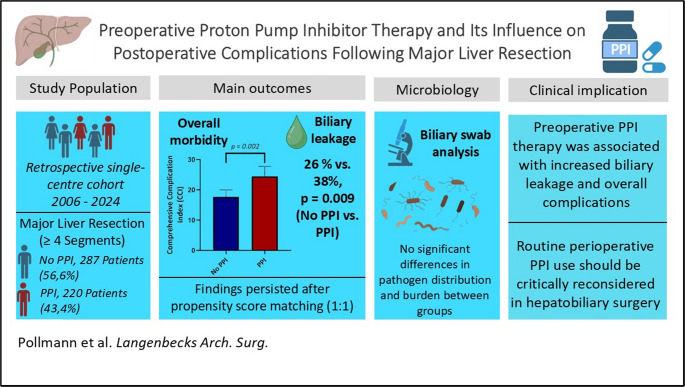

**Supplementary Information:**

The online version contains supplementary material available at 10.1007/s00423-026-04119-x.

## Introduction

### Background

PPI therapy is widely used for the prophylaxis and treatment of gastroduodenal ulcers. For example, stress ulcer prophylaxis with acid-suppressive therapy is indicated in patients requiring prolonged mechanical ventilation (> 48 h) in both medical and surgical intensive care settings [1]. However, the routine use of acid-suppressive therapy has been increasingly questioned in recent years because of emerging evidence linking PPIs to adverse effects, including a higher risk of hospital-acquired pneumonia [2], chronic kidney disease [3] and *Clostridioides difficile* infection [4]. These associations are of particular relevance in hospitalized surgical patients, who are already vulnerable to infectious complications and organ dysfunction.

Beyond promoting *C. difficile* overgrowth, prolonged PPI therapy has been demonstrated to alter the composition of the intestinal microbiome to a considerable extent, reducing microbial diversity and promoting the expansion of opportunistic pathogens [5]. In line with these findings, recent studies suggest an increased risk of postinterventional cholangitis, potentially related to PPI-associated alterations in intestinal and biliary microbial colonization [6–8].

### Rationale and knowledge gap

Despite the fact that clear indications for stress ulcer prophylaxis are largely confined to critically ill patients, perioperative PPI therapy is frequently continued or newly initiated in surgical patients without established risk factors for gastrointestinal bleeding [9]. In addition, PPI therapy started during hospitalization is often continued beyond the acute phase without a clear long-term indication [10]. These considerations are of particular importance in hepatobiliary surgery. Major liver resection involves surgical manipulation of the biliary tract and is associated with a substantial risk of postoperative complications, including biliary leakage and intra-abdominal abscess formation.

Given the growing evidence that PPI therapy alters the intestinal microbiome and may predispose biliary infections after endoscopic or percutaneous biliary interventions [6, 7], it is conceivable that perioperative acid suppression could also influence infectious outcomes after hepatic resection. Nevertheless, the impact of pre- and postoperative PPI therapy on postoperative morbidity in patients undergoing major liver surgery remains to be fully explored.

### Objective

The aim of this study was therefore to evaluate the impact of PPI therapy on postoperative complications following major liver resection, with particular focus on postoperative biliary leakage.

## Materials and methods

This retrospective cohort study investigated patients undergoing major liver resection to evaluate the association between proton pump inhibitor (PPI) therapy and postoperative complications, with particular focus on biliary leakage. Major liver resection was defined as resection of four or more Couinaud liver segments [11]. All patients who underwent major liver resection at our center between 2006 and 2024 were identified after approval by the local ethics committee (ID: 2025-4013-BO-D; approval date: 06.11.2025). The study was further registered in the German Clinical Trials Register (application number: DRKS00038981).

Patients who underwent simultaneous major liver resection and additional surgical procedures (e.g., colorectal or pancreatic resection) were excluded. Major liver resections were performed according to institutional standards by experienced hepatobiliary surgeons. Parenchymal transection was predominantly performed using ultrasonic aspiration technique (CUSA), with selective ligation or clipping of vascular and biliary structures according to operative requirements. Data on pre- and postoperative PPI therapy were collected from medical records; patients with missing information on PPI exposure were excluded from the analysis. The remaining cohort was stratified into two groups: patients receiving preoperative PPI therapy and those without preoperative PPI therapy.

Baseline characteristics were recorded, including age, gender, body mass index (BMI), American Society of Anesthesiologists (ASA) score, underlying diagnosis, preoperative biliary stent placement, type of surgical procedure, biliodigestive anastomosis, and relevant comorbidities. To ensure patient confidentiality, all identifying information was removed prior to analysis, and only data relevant to the study objectives were accessed.

The primary endpoint was overall postoperative morbidity, assessed using the CCI based on the Clavien–Dindo classification [12]. The secondary endpoint was the occurrence of postoperative biliary leakage, which was defined and graded according to the criteria of the International Study Group of Liver Surgery (ISGLS) [13]. In addition, microorganisms identified from sterile swabs obtained from the biliary tree were analyzed and compared between groups.

### Statistical analysis

After data collection, patients with and without preoperative PPI therapy were compared with regard to baseline characteristics. Categorical variables, including gender, preoperative biliary stenting, liver transection technique, presence of biliodigestive anastomosis, and comorbidities, were analyzed using Fisher’s exact test. The ASA score, underlying diagnosis, and type of surgical procedure were compared using the chi-squared test. Continuous variables (age and BMI) were tested for normality and, as they were not normally distributed, were compared using the Mann–Whitney U test. No adjustments were made for multiple comparisons.

The CCI as primary endpoint was also non-normally distributed and was therefore compared between groups using the Mann–Whitney U test. In addition, the number and severity of postoperative complications were analyzed according to the Clavien–Dindo classification using the chi-square test. The occurrence of postoperative biliary leakage was analyzed using Fisher’s exact test. Furthermore, the chi-square test was used to compare the severity of biliary leakage. The number of microorganisms identified in biliary swabs was compared using the Mann–Whitney U test, while the distribution of specific microorganisms was analyzed descriptively. Data are presented as mean ± standard deviation unless otherwise specified.

To improve comparability between groups, propensity score matching was subsequently performed. Matching variables included age, diagnosis, ASA score, and type of surgical procedure. Matching was conducted using the case-control matching tool in MedCalc Statistical Software (MedCalc Software Ltd., Ostend, Belgium), which applies a nearest-neighbor matching approach using a greedy caliper matching algorithm [14, 15]. Age matching was performed within a maximum allowed difference of ± 3 years, while exact matching was required for all other variables. Covariate balance was assessed using standardized mean differences (SMDs), with values < 0.10 considered indicative of acceptable balance. After matching, baseline characteristics, CCI, and the occurrence of biliary leakage were reassessed using the same statistical methods described above.

Furthermore, a multivariate logistic regression analysis was performed to further evaluate whether preoperative PPI therapy was independently associated with postoperative biliary leakage. The analyzed variables included preoperative PPI therapy, biliodigestive anastomosis, preoperative biliary stenting, diagnosis, age, and ASA score. Odds ratios (ORs) with 95% confidence intervals (CIs) were calculated.

All statistical analyses were performed using MedCalc Statistical Software (MedCalc Software Ltd., Ostend, Belgium). Figures were created using GraphPad Prism (GraphPad Software Inc., version 10.4.0, San Diego, CA, USA) and Fiji open-source software. Missing data were not imputed, and analyses were performed using available case data only. All tests were two-tailed, and a p-value < 0.05 was considered statistically significant.

## Results

### Patient characteristics

**Fig. 1:**
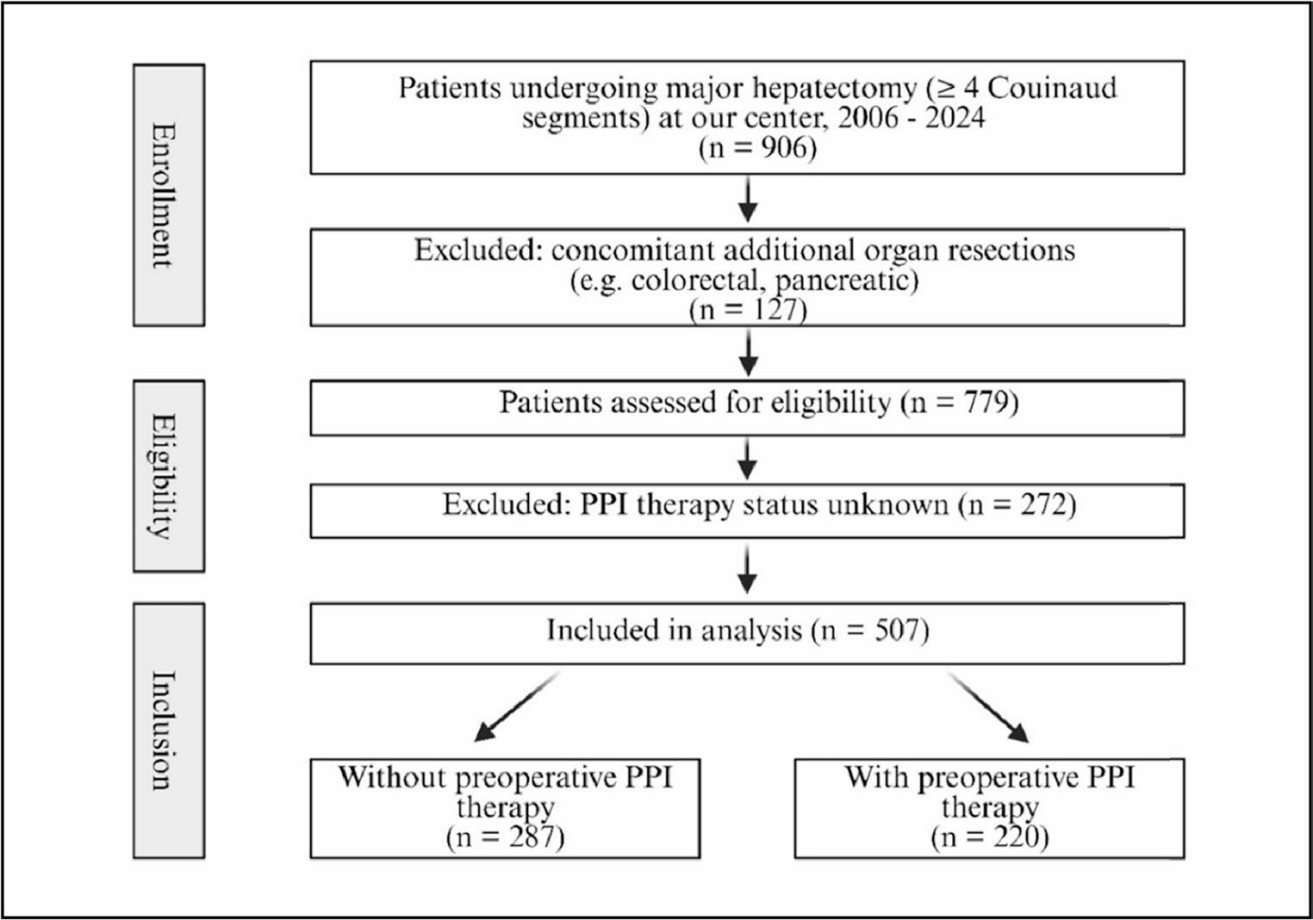
Flow chart illustrating patient selection of the current study

Between 2006 and 2024, a total of 906 major hepatectomies were performed at the university hospital Jena. Of these, 127 procedures were excluded due to concomitant additional surgical resections (e.g., colorectal or pancreatic resections). In a further 272 patients, pre- or postoperative PPI therapy could not be reliably determined retrospectively. Consequently, 507 patients were included in the final analysis (Fig. 1).

Among these, 220 patients (43.4%) received preoperative PPI therapy, whereas 287 patients (56.6%) did not. Overall, 455 of 507 patients (89.7%) received postoperative PPI therapy at hospital discharge. Postoperative PPI use was significantly more common among patients with preoperative PPI exposure, with 214 of 220 patients (97.3%) continuing PPI therapy postoperatively compared to 241 of 287 patients (84.0%) without preoperative PPI therapy (*p* < 0.001).

Baseline characteristics (Age, sex distribution, BMI and ASA score) were comparable between groups (Table 1). Mean age was 63.6 ± 11.8 years in patients with preoperative PPI therapy and 61.7 ± 12.2 years in those without (*p* = 0.053). Female patients accounted for 47% of the PPI group and 51% of the non-PPI group (*p* = 0.147). BMI was marginally lower in the PPI group compared to the non-PPI group (25 ± 4.4 vs. 25.8 ± 4.4 kg/m², *p* = 0.108).

**Table 1:** Patient characteristics

	No preoperative PPI therapy *n* = 287	Preoperative PPI therapy *n* = 220	*P*-value
Gender (% female)	146 (51)	105 (47)	0.147
Age (mean ± SD) in years	61.7 ± 12.2	63.6 ± 11.8	0.053
BMI (mean ± SD) in kg/m² ^†^	25.8 ± 4.4	25 ± 4.4	0.108
Postoperative PPI therapy (%)	**241 (84)**	**214 (97)**	**0.001**
Diabetes mellitus Type II (%)	42/238 (15)	37/220 (17)	0.621
Arterial Hypertension (%)	**172/286 (60)**	**156/220 (71)**	**0.014**
COPD (%)	14/286 (5)	19/220 (9)	0.103
Heart disease (%)	45/286 (16)	32/220 (14)	0.803
Vascular disease (%)	23/286 (8)	28/220 (13)	0.101
Chronic kidney disease (%)	8/286 (3)	8/220 (4)	0.617
Steroid therapy (%)	5/286 (2)	6/220 (3)	0.544
ASA-Grade^‡^			0.350
I (%)	12/227 (5)	5/163 (3)	
II (%)	125/227 (55)	84/163 (51)	
III (%)	90/227 (40)	73/163 (45)	
IV (%)	0/227 (0)	1/163 (1)	
Preoperative biliary stenting (%)	**36 (12)**	**45 (20)**	**0.020**
Diagnosis			**0.018**
Hepatocellular carcinoma	**24 (8)**	**32 (14)**	
Cholangiocarcinoma	**82 (29)**	**72 (33)**	
Gallbladder carcinoma	**11 (4)**	**8 (4)**	
Colorectal metastases	**98 (34)**	**61 (28)**	
Other metastases	**23 (8)**	**26 (12)**	
Non-malignant liver tumor	**49 (17)**	**21 (9)**	
Surgical technique			0.209
Right hemihepatectomy	116 (40)	85 (39)	
Left hemihepatectomy	23 (8)	13 (5)	
Extended right hemihepatectomy	38 (13)	33 (15)	
Extended left hemihepatectomy	14 (5)	17 (8)	
Right trisectorectomy	43 (15)	47 (21)	
ALPPS^§^ procedure	26 (9)	12 (5)	
Left trisectorectomy	25 (9)	13 (6)	
Other	2 (1)	0 (0)	
Parenchymal transection technique			1.000
Ultrasonic aspiration transection (%)	286/287 (100)	217/218 (100)	
Robotic Clamp-crush technique (%)	1/287 (0)	1/218 (0)	
Biliodigestive anastomosis	**68 (24)**	**72 (33)**	**0.027**

†*BMI* Body Mass Index, ‡*ASA* American Society of Anaesthesiologists, § *ALPPS* Associating Liver Partition and Portal vein ligation for Staged hepatectomy

There was a significant difference in the underlying diagnosis leading to liver resection between groups (*p* = 0.018). Notably, cholangiocarcinoma was more frequent in the PPI group, whereas colorectal liver metastases predominated among patients without preoperative PPI therapy. Hepatocellular carcinoma was also more common in patients receiving PPI therapy.

Furthermore, preoperative biliary stenting was more often performed in patients with preoperative PPI therapy compared to those without (20% vs. 12%, respectively, *p* = 0.020).

The distribution of surgical procedures did not differ significantly between groups (*p* = 0.209). Right hemihepatectomy was the most common procedure in both groups (40% vs. 39%), followed by extended right hemihepatectomy and right trisectorectomy. A higher proportion of right trisectorectomies was observed in the PPI group (21% vs. 15%), although this difference did not reach statistical significance. No differences in parenchymal transection techniques were observed between groups, with ultrasonic aspiration transection (CUSA) used in nearly all patients (99.7% vs. 99.5%, *p* = 1.000).

Importantly, biliodigestive anastomoses were performed significantly more often in patients receiving preoperative PPI therapy compared with those who did not (33% vs. 24%, *p* = 0.027).

Comorbidities such as diabetes mellitus, heart disease, chronic kidney disease, and preoperative steroid therapy were similarly distributed between groups. However, arterial hypertension (71% vs. 60%, *p* = 0.014), chronic obstructive pulmonary disease (COPD) (9% vs. 5%, *p* = 0.103), and vascular disease (13% vs. 8%, *p* = 0.101) were more prevalent among patients receiving preoperative PPI therapy.

In summary, while age, sex, ASA score, and type of liver resection were comparable between groups, patients receiving preoperative PPI therapy more frequently presented with cholangiocarcinoma, preoperative stenting, underwent biliodigestive reconstruction, and exhibited a higher prevalence of cardiovascular comorbidities.

### Postoperative overall complications and biliary leakage

**Fig. 2:**
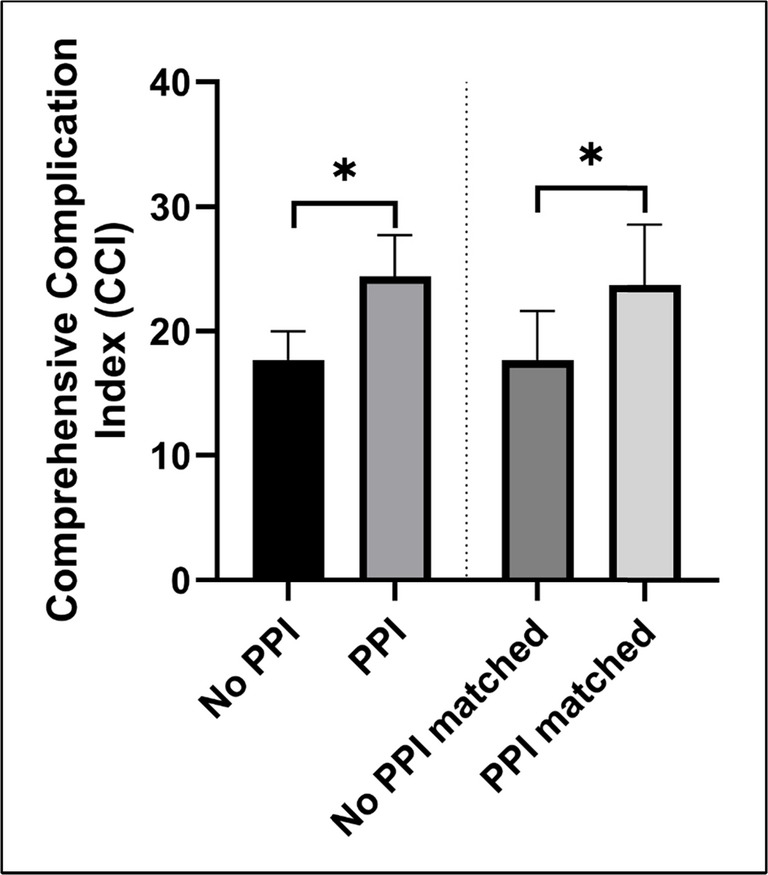
Postoperative complications stratified by the Comprehensive Complication Index (CCI) in patients without and with preoperative PPI therapy, before and after propensity score matching. Mean values and 95% confidence intervals are shown. A single asterisk (*) indicates *P* < 0.05

Overall postoperative complications including biliary leakage, as the most relevant complication after major liver resection, were analyzed. Group comparison demonstrated a significantly higher CCI in patients receiving preoperative PPI therapy compared with those without preoperative PPI therapy (24.4 ± 25 vs. 17.7 ± 19.7, *p* = 0.002), see Fig. 2.

Analysis according to the Clavien–Dindo classification showed that minor complications (Grade I) were slightly but significantly more frequent in patients with preoperative PPI therapy compared to those without (41% vs. 33%, *p* = 0.049). In addition, Grade IIIb complications requiring surgical treatment occurred more often in patients receiving preoperative PPI therapy (14% vs. 8%, *p* = 0.040). Postoperative mortality was significantly higher in patients with preoperative PPI therapy compared with patients without preoperative PPI therapy (5% vs. 2%, *p* = 0.043).

**Fig. 3:**
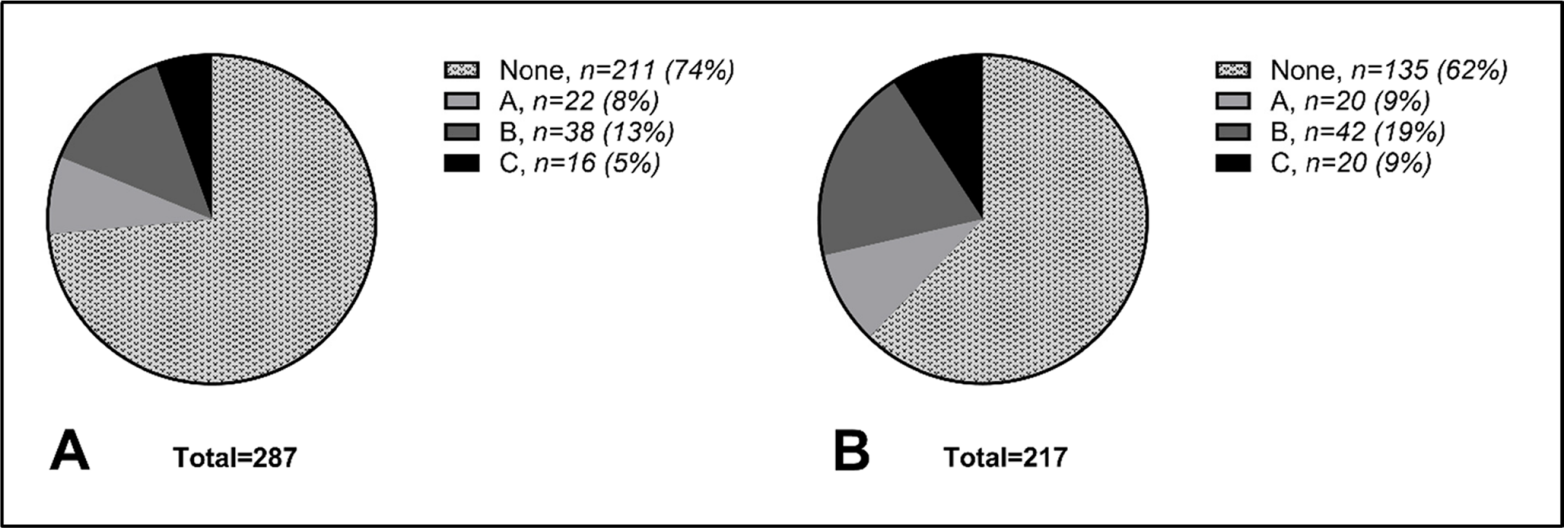
Severity of biliary leakage according to the International Study Group of Liver Surgery (ISGLS) grading in patients without (**A**) and with (**B**) preoperative PPI therapy

The incidence of postoperative biliary leakage was significantly higher in patients receiving preoperative PPI therapy than in those without preoperative PPI therapy (38% vs. 26%, *p* = 0.009). Stratification by ISGLS grade demonstrated a slightly but significantly increased rate of biliary leakage grades A, B, and C in patients with preoperative PPI therapy (Fig. 3). The results are summarized in Table 2. Furthermore, a descriptive analysis across predefined study intervals was performed to assess potential temporal changes. While preoperative PPI use remained stable over time (41–46%), postoperative PPI use and biliary leakage rates increased throughout the study period (Supplementary Table S1).

**Table 2:** Results

	No PPI therapy *n* = 287	PPI therapy *n* = 220	*P*-value
CCI^†^ (mean ± SD)	17.7 ± 19.7	24.4 ± 25	0.002
Clavien-Dindo 1 (%)	**93/285 (33)**	**89/215 (41)**	**0.049**
Clavien-Dindo 2 (%)	101/285 (35)	71/215 (33)	0.635
Clavien-Dindo 3a (%)	59/285 (21)	60/215 (28)	0.071
Clavien-Dindo 3b (%)	**23/285 (8)**	**30/215 (14)**	**0.040**
Clavien-Dindo 4a (%)	11/285 (4)	11/215 (5)	0.516
Clavien-Dindo 4b (%)	1/285 (0)	3/215 (1)	0.319
Clavien-Dindo 5 (%)	**5/287 (2)**	**11/220 (5)**	**0.043**
Biliary leakage	**76/287 (26)**	**82/217 (38)**	**0.009**
Severity of biliary leakage			**0.049**
Grade A (%)	**22/287 (8)**	**20/217 (9)**	
Grade B (%)	**38/287 (13)**	**42/217 (19)**	
Grade C (%)	**16/287 (6)**	**20/217 (9)**	
Propensity-Score matching	*n* = 98	*n* = 98	
CCI^†^ (mean ± SD)	**17.7 ± 19.5**	**23.7 ± 24.1**	**0.047**
Clavien-Dindo 1 (%)	29/96 (30)	41/94 (44)	0.071
Clavien-Dindo 2 (%)	29/96 (30)	28/94 (30)	1.000
Clavien-Dindo 3a (%)	20/96 (21)	28/94 (30)	0.183
Clavien-Dindo 3b (%)	8/96 (8)	12/94 (12)	0.353
Clavien-Dindo 4a (%)	4/96 (4)	6/94 (6)	0.534
Clavien-Dindo 4b (%)	0/96 (0)	1/94 (1)	0.495
Clavien-Dindo 5 (%)	2/98 (2)	4/98 (4)	0.683
Biliary leakage	**26/98 (27)**	**40/97 (41)**	**0.035**
Severity of biliary leakage			**0.018**
Grade A (%)	**11/98 (11)**	**11/97 (11)**	
Grade B (%)	**10/98 (10)**	**20/97 (21)**	
Grade C (%)	**5/98 (5)**	**9/97 (9)**	

†*CCI* Comprehensive Complication Index

### Postoperative overall complications and biliary leakage after propensity score matching

Given the increased baseline risk profile observed in patients receiving preoperative PPI therapy, propensity score matching was performed to improve comparability between cohorts. After matching, patients showed largely overlapping baseline characteristics, see Table S2 of the supplementary appendix. No statistically significant differences were observed between the matched groups, each comprising 98 patients. Furthermore, assessment of standardized mean differences (SMDs) before and after matching demonstrated acceptable balance for all predefined matching variables, with all post-matching SMDs below 0.10, see supplementary Table S3. However, diabetes mellitus was slightly more frequent in patients without preoperative PPI therapy (22% vs. 18%, *p* = 0.595), whereas arterial hypertension was more prevalent among patients with preoperative PPI therapy (76% vs. 65%, *p* = 0.159). In addition, preoperative biliary stenting was more common in patients receiving preoperative PPI therapy, although this difference did not reach statistical significance (22% vs. 15%, *p* = 0.208). Intraoperatively, biliodigestive anastomosis was performed more frequently in patients with preoperative PPI therapy compared with those without, again without reaching statistical significance (34% vs. 25%, *p* = 0.208).

After matching, overall postoperative morbidity remained significantly higher in patients receiving preoperative PPI therapy compared with those without preoperative PPI therapy, as reflected by a higher CCI (23.7 ± 24.1 vs. 17.7 ± 19.5, *p* = 0.047), see Fig. 2. Stratification by Clavien–Dindo classification showed a trend toward higher rates of Grade I and Grade IIIa complications in patients with preoperative PPI therapy, although these differences were not statistically significant.

Postoperative biliary leakage occurred significantly more often in patients receiving preoperative PPI therapy than in those without (41% vs. 27%, *p* = 0.035). Severity analysis revealed that Grade B and C biliary leakages, requiring therapeutic interventions including relaparotomy (Grade C), were more frequent in patients with preoperative PPI therapy. In contrast, the rate of Grade A biliary leakage, which did not require changes in clinical management, was similar between both groups (Figure S1 of the supplementary material).

### Multivariable logistic regression analysis of risk factors for postoperative biliary leakage

In addition, a multivariable logistic regression analysis was performed to further evaluate whether preoperative PPI therapy was independently associated with postoperative biliary leakage after adjustment for clinically relevant confounders. The model included preoperative PPI therapy, biliodigestive anastomosis, preoperative biliary stenting, diagnosis, age, and ASA score as covariates. Preoperative PPI therapy remained independently associated with an increased risk of postoperative biliary leakage (OR 1.95, 95% CI 1.21–3.14, *p* = 0.006). Furthermore, biliodigestive anastomosis was identified as an independent risk factor for biliary leakage (OR 2.80, 95% CI 1.38–5.67, *p* = 0.004). In contrast, preoperative biliary stenting was not independently associated with postoperative biliary leakage after adjustment for the included covariates. Increasing age was also associated with a higher risk of biliary leakage, whereas diagnosis and ASA score were not significantly associated with the occurrence of biliary leakage in the adjusted analysis. The results are summarized in Table 3.

**Table 3:** Multivariable logistic regression analysis of risk factors for postoperative biliary leakage

Variable	Odds ratio	95% Confidence interval	*P*-value
Age	**1.03**	**1.01–1.05**	**0.012**
ASA – Grade			
I	0.82	0.22–3.13	0.774
III	1.04	0.63–1.70	0.890
Biliodigestive anastomosis	**2.80**	**1.38–5.67**	**0.004**
Preoperative PPI therapy	**1.95**	**1.21–3.14**	**0.006**
Diagnosis			
Hepatocellular carcinoma	**0.23**	**0.08–0.73**	**0.012**
Cholangiocarcinoma	1.47	0.71–3.03	0.294
Gallbladder carcinoma	0.54	0.16–1.85	0.330
Other metastases	1.99	0.85–4.66	0.113
Non-malignant liver tumor	1.44	0.60–3.49	0.413
Preoperative biliary stenting	1.17	0.56–2.42	0.678

### Microbiological characterization from biliary swabs

In a selected subgroup of patients, microbiological analysis of the biliary system was available from a total of 126 sterile swabs, with one swab obtained per patient. The overall number of identified microorganisms was comparable between patients with and without preoperative PPI therapy (1.5 ± 1.5 vs. 1.9 ± 1.7, respectively; *p* = 0.390), and no distinct pathogen pattern associated with preoperative PPI use was observed.

In contrast, preoperative biliary stenting emerged as the predominant factor influencing microbial burden within the biliary tree. Patients who underwent preoperative biliary stenting exhibited a markedly higher number of identified microorganisms compared with patients without preoperative stenting (2.8 ± 1.5 vs. 0.7 ± 1.0). Across both study groups, the detected microorganisms mainly comprised typical biliary and enteric flora, including *Enterococcus* species, enteric Gram-negative bacteria, and *Candida* species, without relevant qualitative differences between patients with and without preoperative PPI therapy.

## Discussion

### Key findings

In this retrospective cohort study of patients undergoing major liver resection, preoperative PPI therapy was associated with increased postoperative morbidity, as reflected by a higher CCI and a significantly increased incidence of biliary leakage. Importantly, these associations persisted after propensity score matching for relevant baseline and surgical characteristics as well as multivariable adjustment for established biliary risk factors, suggesting that preoperative PPI therapy may represent an independent marker of increased postoperative risk in this population.

Biliary leakage remains one of the most clinically relevant complications after major liver resection and is a key driver of postoperative morbidity, prolonged hospitalization, and infectious complications [16]. In the present study, patients receiving preoperative PPI therapy demonstrated a consistently higher rate of biliary leakage, particularly of clinically relevant ISGLS grade B and C leaks requiring interventional or surgical management [17, 18]. These findings are of particular interest given the high prevalence of perioperative PPI use and the absence of clear guideline-based indications for stress ulcer prophylaxis in most elective hepatobiliary surgical patients [19].

### Explanation of findings

The mechanisms underlying the observed association between PPI therapy and postoperative complications require further investigation. PPIs are known to markedly reduce gastric acidity and to alter the intestinal microbial composition [5]. Consistent with this, several studies have demonstrated that PPI therapy is associated with an increased susceptibility to infectious complications, including hospital-acquired pneumonia [2] and *Clostridioides difficile* infection [20]. In addition, recent evidence indicates that bile is not sterile and that alterations in intestinal microbiota may influence biliary colonization and infection, particularly after biliary interventions [6, 7].

In the present study, microbiological analysis of biliary swabs was performed in a subgroup of patients. No significant differences in microbial burden or pathogen distribution were observed between patients with and without preoperative PPI therapy. In contrast, preoperative biliary stenting emerged as the predominant determinant of biliary microbial load in this subgroup, most likely due to biofilm formation and ascending bacterial colonization. This finding is consistent with previous reports identifying biliary stenting as a major driver of biliary contamination and infection risk [6, 7].

However, the absence of clear microbiological differences does not preclude a clinically relevant effect of PPI therapy. First, microbiological data were available only in a limited subset of patients. Second, the analysis was based on biliary swabs, which provide only a partial view of biliary microbiology; previous studies have shown that bile duct aspirates yield a higher detection rate of pathogens, particularly anaerobic bacteria [21]. Moreover, differentiation between bacterial colonization and true infection was not possible in the current study.

However, the absence of a distinct pathogen pattern in the present cohort suggests that additional mechanisms may be involved. In line with this, experimental and clinical studies indicate that PPI therapy may influence several biological pathways beyond gastric acid suppression. PPI exposure has been associated with alterations in intestinal barrier integrity and increased mucosal permeability, potentially facilitating translocation of microbial products [22]. Furthermore, PPI therapy has been linked to modulation of innate immune responses, including altered neutrophil activity and changes in inflammatory signaling pathways that may influence postoperative host responses [23, 24]. Beyond effects on gut microbiota and intestinal barrier integrity, experimental studies suggest that chronic PPI exposure may alter bile acid signaling and biliary epithelial homeostasis, potentially affecting tissue repair mechanisms [25]. While these mechanisms remain speculative in the context of the present study, they provide a biologically plausible framework for the observed association and support further prospective and mechanistic investigations.

### Strengths and limitations

A central strength of this study is the comparatively large single-center cohort of patients undergoing major liver resection with detailed perioperative clinical data. Relevant patient characteristics and surgical parameters were comprehensively collected and incorporated into the analysis, including standardized outcome measures such as the CCI [12] and ISGLS defined biliary leakage [13]. In addition to propensity score matching, a multivariable logistic regression model incorporating established biliary risk factors was performed to further address residual confounding. Nevertheless, several limitations warrant consideration. The retrospective design precludes causal inference, and residual confounding cannot be fully excluded despite propensity score matching. In particular, perioperative antibiotic regimens could not be reliably reconstructed and therefore could not be incorporated into the analysis, although antibiotic selection is known to influence postoperative infectious outcomes in hepatobiliary surgery [26]. In addition, patients receiving preoperative PPI therapy more frequently presented with cholangiocarcinoma, biliary reconstruction, and preoperative biliary stenting, all of which are recognized risk factors for postoperative biliary complications [27]. In line with this, preoperative PPI therapy may represent a surrogate marker for increased comorbidity burden and polypharmacy [28], both of which have been associated with adverse postoperative outcomes in broader surgical populations. However, the association between preoperative PPI therapy and biliary leakage persisted after multivariable adjustment for these variables, suggesting that the observed association was not solely attributable to differences in baseline characteristics. Temporal analysis demonstrated relatively stable preoperative PPI use rates over time, whereas postoperative PPI use increased from 83% to 94%. Likewise, biliary leakage rates increased from 24% to 35% across the study period. However, these findings may reflect temporal changes in patient comorbidity and surgical complexity rather than a direct association between PPI exposure and postoperative outcomes. Furthermore, detailed information regarding the timing, duration, and specific indications for PPI therapy was not uniformly available, and the relatively high exclusion rate due to missing information on preoperative PPI intake represents an additional limitation.

Importantly, the impact of postoperative PPI therapy on biliary leakage and overall postoperative morbidity was not specifically analyzed, as 89.7% of patients received PPI therapy at hospital discharge. Postoperative PPI therapy was significantly more common among patients with preoperative PPI exposure and may therefore represent an additional source of residual confounding. Given the substantial overlap between preoperative and postoperative PPI exposure, the independent effect of postoperative PPI therapy on postoperative outcomes could not be adequately assessed in the present study. Current recommendations suggest stress ulcer prophylaxis primarily for critically ill patients and selected high-risk constellations [29]. Previous studies have additionally emphasized the importance of discontinuing unnecessary PPI therapy after intensive care treatment to avoid unintended long-term use [10]. Therefore, the potential influence of postoperative PPI exposure on postoperative outcomes should be specifically addressed in future prospective studies. Finally, the single-center design may limit external validity, as institutional differences in patient selection, operative techniques, and perioperative management may influence postoperative outcomes. Confirmation of these findings in multicenter investigations is therefore warranted.

Despite these limitations, this study provides clinically relevant insights into the potential impact of preoperative PPI therapy in major liver surgery.

### Implications and actions needed

Given the widespread use of PPIs and the lack of clear indications in most elective surgical patients, our findings underscore the need for careful perioperative evaluation of acid-suppressive therapy. Prospective studies are warranted to further elucidate underlying mechanisms and to determine whether restrictive or indication-based PPI strategies may reduce postoperative morbidity after major hepatobiliary surgery.

## Conclusions

Preoperative PPI therapy was associated with increased postoperative morbidity and a higher incidence of biliary leakage after major liver resection. These associations persisted after adjustment for baseline risk factors, suggesting that perioperative PPI therapy may represent a relevant and potentially modifiable risk marker in hepatobiliary surgery. Given the observational design of the present study, these findings should be considered hypothesis-generating and do not establish a causal relationship. While patients with a clear clinical indication for PPI therapy should continue to receive appropriate treatment, careful evaluation of perioperative PPI use in patients without a well-established indication may be warranted. Further prospective studies are needed to clarify the potential impact of perioperative PPI exposure on postoperative outcomes.

## Supplementary Information

Below is the link to the electronic supplementary material.

Supplementary Material 1 (DOCX 190 KB)

## Data Availability

De-identified participant data underlying this study will be available from publication for 5 years upon reasonable request to the corresponding author.

## Declarations

### Previous communication

This research is not based on a previous communication.

### Utilization of artificial intelligence

No artificial intelligence tool was used for the preparation of our manuscript.
