# Supplementary material for: Preoperative proton pump inhibitor therapy and its influence on postoperative complications following major liver resection
Source: Langenbecks Arch Surg. 2026 Jun 27;411(1):173. doi: 10.1007/s00423-026-04119-x (PMC13315170; doi:10.1007/s00423-026-04119-x)
Supplement: Supplementary file 1 — Supplementary Material 1 (DOCX 190 KB) [file 423_2026_4119_MOESM1_ESM.docx]

**Supplementary Appendix**

**Table S1.** Temporal trends in PPI usage and biliary leakage across the study period

| Study period | Number of patients (%) | Preoperative PPI therapy (%) | Postoperative PPI therapy (%) | Biliary leakage (%) |
| --- | --- | --- | --- | --- |
| 2006 – 2011 | 134 (26) | 55 (41) | 111 (83) | 32 (24) |
| 2012 – 2018 | 244 (48) | 112 (46) | 223 (91) | 81 (33) |
| 2019 – 2024 | 129 (25) | 53 (41) | 121 (94) | 45 (35) |

**Table S2.** Patient characteristics of matched cohorts

|  | No PPI therapy  n = 98 | PPI therapy  n = 98 | P-value |
| --- | --- | --- | --- |
| Gender (% female) | 48 (49) | 46 (47) | 0.886 |
| Age (mean ± SD) in years | 64.9 ± 9.6 | 65.1 ± 9.7 | 0.848 |
| BMI (mean ± SD) in kg/m²^†^ | 26.7 ± 4.4 | 26.3 ± 4.2 | 0.627 |
| Diabetes mellitus Type II (%) | 21/97 (22) | 18/98 (18) | 0.595 |
| Arterial Hypertension (%) | 64 (65) | 74 (76) | 0.159 |
| COPD (%) | 5 (5) | 9 (9) | 0.406 |
| Heart disease (%) | 14 (14) | 19 (19) | 0.446 |
| Vascular disease (%) | 13 (13) | 14 (14) | 0.580 |
| Chronic kidney disease (%) | 4 (4) | 5 (5) | 1.000 |
| Steroid therapy (%) | 0 (0) | 2 (2) | 0.497 |
| ASA-Grade^‡^ |  |  | 1.000 |
| I (%) | 2/79 (2) | 2/79 (2) |  |
| II (%) | 40/79 (41) | 40/79 (41) |  |
| III (%) | 37/79 (38) | 37/79 (38) |  |
| IV (%) | 0/79 (0) | 0/79 (0) |  |
|  |  |  |  |
| Preoperative biliary stenting (%) | 15 (15) | 22 (22) | 0.208 |
| Diagnosis |  |  | 1.000 |
| Hepatocellular carcinoma | 7 (7) | 7 (7) |  |
| Cholangiocarcinoma | 35 (36) | 35 (36) |  |
| Gallbladder carcinoma | 2 (2) | 2 (2) |  |
| Colorectal metastases | 39 (40) | 39 (40) |  |
| Other metastases | 6 (6) | 6 (6) |  |
| Non-malignant liver tumour | 9 (9) | 9 (9) |  |
|  |  |  |  |
| Surgical technique | |  | 1.000 |
| Right hemihepatectomy | 43 (44) | 43 (44) |  |
| Left hemihepatectomy | 13 (13) | 13 (13) |  |
| Extended right hemihepatectomy | 9 (9) | 9 (9) |  |
| Extended left hemihepatectomy | 4 (4) | 4 (4) |  |
| Right trisectorectomy | 16 (16) | 16 (16) |  |
| ALPPS^§^ procedure | 5 (5) | 5 (5) |  |
| Left trisectorectomy | 8 (8) | 8 (8) |  |
|  |  |  |  |
| Biliodigestive anastomosis | 24 (25) | 33 (34) | 0.208 |

† BMI = Body Mass Index; ‡ ASA = American Society of Anaesthesiologists; § ALPPS = Associating Liver Partition and Portal vein ligation for Staged hepatectomy

**Table S3.** Standardized mean differences before and after matching for predefined matching variables

| Variable | Standardized mean differences before matching | Standardized mean differences after matching |
| --- | --- | --- |
| Age | 0.17 | 0.024 |
| ASA-Grade | 0.111 | 0.000 |
| Diagnosis | 0.223 | 0.000 |
| Surgical technique | 0.166 | 0.000 |

**Table S4.** Baseline characteristics of patients with known and unknown PPI status

|  | Known PPI status  n = 507 | Unknown PPI status  n = 272 | P-value |
| --- | --- | --- | --- |
| Gender (% female) | 251 (49) | 128 (47) | 0.55 |
| Age (mean ± SD) in years | 62.0 ± 12.1 | 61.6 ± 11.7 | 0.65 |
| BMI (mean ± SD) in kg/m² ^†^ | 25.5 ± 4.4 | 25.8 ± 5.2 | 0.38 |
| Diabetes mellitus Type II (%) | 79/458 (17) | 48/265 (18) | 0.76 |
| Arterial Hypertension (%) | 328/506 (65) | 154/245 (63) | 0.56 |
| COPD (%) | 33/506 (7) | 13/252 (5) | 0.39 |
| Heart disease (%) | 77/506 (15) | 35/248 (14) | 0.72 |
| Vascular disease (%) | 51/506 (10) | 23/250 (9) | 0.75 |
| Chronic kidney disease (%) | 16/506 (3) | 10/252 (4) | 0.61 |
| Steroid therapy (%) | 11/506 (2) | 7/220 (3) | 0.57 |
| **ASA-Grade^‡^** |  |  | **0.04** |
| **I (%)** | **17/390 (4)** | **20/225 (9)** |  |
| **II (%)** | **209/390 (54)** | **119/225 (53)** |  |
| **III (%)** | **163/390 (42)** | **86/225 (38)** |  |
| **IV (%)** | **0/227 (0)** | **0/225 (0)** |  |
|  |  |  |  |
| Preoperative biliary stenting (%) | 81 (16) | 32 (14) | 0.51 |
| Diagnosis |  |  | 0.09 |
| Hepatocellular carcinoma | 56 (11) | 24 (9) |  |
| Cholangiocarcinoma | 154 (30) | 60 (22) |  |
| Gallbladder carcinoma | 19 (4) | 8 (3) |  |
| Colorectal metastases | 159 (31) | 95 (35) |  |
| Other metastases | 49 (10) | 33 (12) |  |
| Non-malignant liver tumor | 70 (14) | 52 (19) |  |
|  |  |  |  |
| Surgical technique | |  | 0.89 |
| Right hemihepatectomy | 201 (40) | 114 (42) |  |
| Left hemihepatectomy | 36 (8) | 24 (9) |  |
| Extended right hemihepatectomy | 71 (14) | 33 (12) |  |
| Extended left hemihepatectomy | 31 (6) | 16 (6) |  |
| Right trisectorectomy | 90 (18) | 44 (16) |  |
| ALPPS^§^ procedure | 38 (7) | 22 (8) |  |
| Left trisectorectomy | 38 (7) | 19 (7) |  |
| Other | 2 (0) | 0 (0) |  |
|  |  |  |  |
| Biliodigestive anastomosis | 140 (28) | 65 (24) | 0.23 |

† BMI = Body Mass Index; ‡ ASA = American Society of Anaesthesiologists; § ALPPS = Associating Liver Partition and Portal vein ligation for Staged hepatectomy

**Figure S1.**


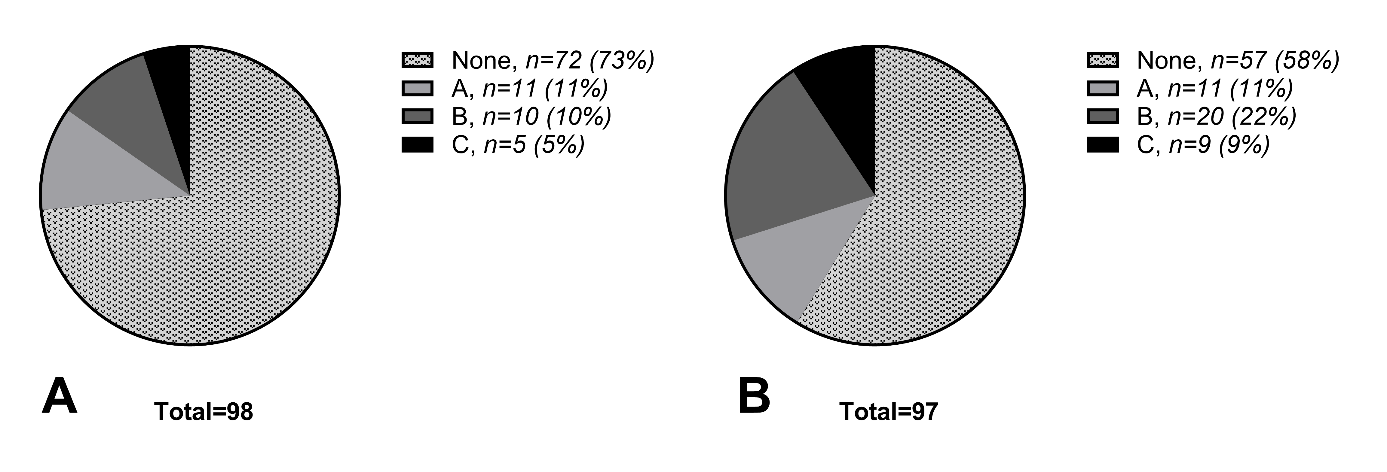


**Figure S1.** Severity of biliary leakage according to the International Study Group of Liver Surgery (ISGLS) grading in propensity score–matched cohorts without (A) and with (B) preoperative PPI therapy.
